# Evaluating sequence data quality from the Swift Accel-Amplicon CFTR Panel

**DOI:** 10.1038/s41597-019-0339-4

**Published:** 2020-01-08

**Authors:** Marco L. Leung, Deborah J. Watson, Courtney N. Vaccaro, Fernanda Mafra, Adam Wenocur, Tiancheng Wang, Hakon Hakonarson, Avni Santani

**Affiliations:** 1 Center for Applied Genomics, Children’s Hospital of Philadelphia, Philadelphia, Pennsylvania USA; 2 Department of Pediatrics, University of Pennsylvania Perelman School of Medicine, Philadelphia, Pennsylvania USA; 3 Department of Pathology and Laboratory Medicine, University of Pennsylvania Perelman School of Medicine, Philadelphia, Pennsylvania USA

**Keywords:** Genetic testing, Next-generation sequencing

## Abstract

Cystic fibrosis (CF) is one of the most common genetic diseases worldwide with high carrier frequencies across different ethnicities. Next generation sequencing of the cystic fibrosis transmembrane conductance regulator (*CFTR*) gene has proven to be an effective screening tool to determine carrier status with high detection rates. Here, we evaluate the performance of the Swift Biosciences Accel-Amplicon CFTR Capture Panel using *CFTR*-positive DNA samples. This assay is a one-day protocol that allows for one-tube reaction of 87 amplicons that span all coding regions, 5′ and 3′UTR, as well as four intronic regions. In this study, we provide the FASTQ, BAM, and VCF files on seven unique *CFTR*-positive samples and one normal control sample (14 samples processed including repeated samples). This method generated sequencing data with high coverage and near 100% on-target reads. We found that coverage depth was correlated with the GC content of each exon. This dataset is instrumental for clinical laboratories that are evaluating this technology as part of their carrier screening program.

## Abstract (summary)

**Table:** 

Measurement(s)	amplicon sequencing
Technology Type(s)	DNA sequencing
Factor Type(s)	CFTR mutations
Sample Characteristic - Organism	Homo sapiens

Machine-accessible metadata file describing the reported data: 10.6084/m9.figshare.10060244

## Background & Summary

Cystic fibrosis (CF) is considered one of the most common genetic diseases, affecting 1 in 2500–3500 live births in Caucasian populations^1^. Over 1500 mutations have been previously reported in the *CFTR* gene. Due to the high carrier rates, the American College of Obstetricians and Gynecologists (ACOG) suggests CF carrier testing for all women who are considering pregnancy or are currently pregnant^2–4^. In 2004, the American College of Medical Genetics and Genomics (ACMG) published a guideline on testing 23 *CFTR* mutations with high carrier frequencies across different ethnicities^3^. However, to increase the detection rate, it has become a common practice for clinical laboratories to expand the *CFTR* panel to more than 100 mutations, and even full gene analysis^5–7^.

In the past three decades, the detection of *CFTR* mutations has evolved through various molecular methods, including reverse dot blot, restriction fragment length polymorphism (RFLP), and Sanger sequencing^8,9^. The advent of next generation sequencing (NGS) leads to a higher clinical sensitivity by screening more targeted *CFTR* mutations and sequencing of the exonic gene regions, as well as a higher throughput by multiplexing many samples into one sequencing run^10,11^. While NGS excels at generating large amount of data, it is time-consuming and less cost-effective for sequencing few targets and low volume of samples. Recently, Swift Biosciences released a pre-designed amplicon/library preparation kit that can amplify the *CFTR* gene using 87 amplicons in one reaction. Combined with Illumina MiSeq Nano kit v2 (300-cycles), this protocol allows for quick turnaround time, low sample volume, and cost effectiveness.

While a previous study had demonstrated that this method could detect frequent and rare *CFTR* mutations when compared to other methods, the technical specifications were not analysed^12^. Here we examine the Accel-Amplicon CFTR Panel using CF-positive samples by assessing the performance of this assay. We processed seven CF-positive samples that represent across the *CFTR* mutation spectrum (missense, nonsense, splicing and indels), and these mutations are recommended in the ACMG guideline^3^. The first run included one normal sample and three CF-positive samples, and the second run included all samples from the first run, with additional four CF-positive samples (Table 1).

**Table 1:** Coverage statistics by samples.

Run:	1	1	1	1	Run 1 Average	2	2	2	2	2	2	2	2	2	2	Run 2 Average
Sample name:	Sample 1	Sample 2	Sample 3	Sample 4		Sample 1	Sample 2	Sample 3	Sample 4-1	Sample 4-2	Sample 4-3	Sample 5	Sample 6	Sample 7	Sample 8	
Read % on target:	98.32	98.38	98.51	98.31	98.38	99.26	99.24	99.29	99.26	99.24	99.21	99.25	99.20	99.20	99.14	99.23
Mean coverage depth	3845	4013	3598	11553	5752	992	766	1680	1647	1356	1473	1405	1340	1438	1344	1344
% of targeted region >20x	100.00	100.00	100.00	100.00	100.00	99.73	99.73	99.73	99.73	99.73	99.73	99.71	99.71	99.71	99.73	99.72
Number of reads	338244	351590	321450	1019024	507577	90686	70252	154784	148982	124390	135350	129530	123800	132844	122072	123269

**Fig. 1:**
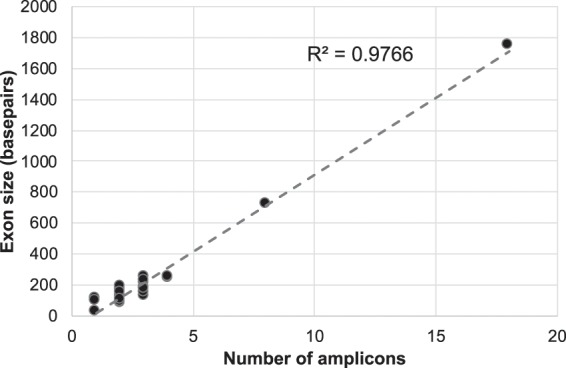
Correlation of amplicon numbers and exon size. The numbers of amplicons for each exon is plotted against the exon size, except intron 1, 12, and 22. A trendline is plotted from the data and R^2^ is calculated to be 0.9766.

Using the MiSeq Nano v2 kit, the sequencing coverage depth averages for run 1 (four samples) and run 2 (ten samples) are 5753x and 1344x, respectively, with almost 100% of the *CFTR* target region being more than 20x (Table 1). As expected for amplicon sequencing, 98–99% of sequencing reads are on-target. We analysed the sequencing performance on the exon level. The coding region, 5′UTR and 3′UTR of the *CFTR* gene has 6123 bp, while the amplicon covers these regions with more than 3000 bp padded region (targeted amplicon size = 9666 bp), with additional amplicons covering four intronic regions (introns 1, 12, 22, and 25) (Table 2). The number of amplicons for each exon correlates with the size of the exons (R^2^ = 0.9766%) (Fig. 1).

**Table 2:** Coverage statistics by exons.

Exon	Legacy exons	# of amplicons	chrom	start	end	amplicon size	exon size	%GC per exon	Mean coverage in run 1	Mean coverage in run 2
5′UTR/exon 1	5′UTR/exon 1	3	7	117119962	117120276	315	185	49.06	6167	1614
intron 1	intron 1	1	7	117138316	117138397	82	n/a	n/a	2336	1241
exon 2	exon 2	1	7	117144280	117144470	191	111	41.44	1938	318
exon 3	exon 3	2	7	117149053	117149317	265	109	35.78	2649	609
exon 4	exon 4	3	7	117170885	117171193	309	216	43.06	10523	2370
exon 5	exon 5	2	7	117174257	117174547	291	90	34.44	2802	677
exon 6	exon 6a	2	7	117175242	117175522	281	164	51.22	9552	2095
exon 7	exon 6b	2	7	117176547	117176786	240	126	36.51	3840	1087
exon 8	exon 7	4	7	117180106	117180469	364	247	44.13	6255	1518
exon 9	exon 8	2	7	117182001	117182229	229	93	36.56	1558	352
exon 10	exon 9	2	7	117188640	117188881	242	183	38.8	2021	377
exon 11	exon 10	2	7	117199456	117199739	284	192	38.54	3458	707
exon 12	exon 11	1	7	117227747	117227914	168	95	42.11	8258	1781
intron 12	intron 11	2	7	117229400	117229594	195	n/a	n/a	2062	730
exon 13	exon 12	2	7	117230379	117230552	174	87	28.74	2472	685
exon 14	exon 13	8	7	117231914	117232756	843	724	40.88	8044	1770
exon 15	exon 14a	3	7	117234856	117235173	318	129	37.98	2200	572
exon 16	exon 14b	1	7	117242841	117242978	138	28	52.63	10428	3268
exon 17	exon 15	3	7	117243554	117243887	334	251	41.04	11254	2708
exon 18	exon 16	2	7	117246632	117246865	234	80	37.5	2422	653
exon 19	exon 17a	2	7	117250542	117250813	272	151	39.07	3944	901
exon 20	exon 17b	3	7	117251517	117251995	479	228	40.79	3318	660
exon 21	exon 18	1	7	117254609	117254804	196	101	42.57	3741	691
exon 22	exon 19	4	7	117267539	117267885	347	250	42.97	7265	1831
intron 22	intron 19	1	7	117279950	117280047	98	n/a	n/a	4677	1872
exon 23	exon 20	3	7	117282467	117282755	289	156	44.87	7179	1671
exon 24	exon 21	2	7	117292796	117293076	281	90	32.22	2681	510
exon 25/intron 25	exon 22/intron 22	3	7	117304586	117304966	381	173	49.71	9076	2349
exon 26	exon 23	2	7	117305458	117305793	336	106	34.91	5435	831
exon 27/3′UTR	exon 24/3′UTR	18	7	117306891	117308755	1865	1758	52.24	6876	1555

Using the manufacturer’s recommended bioinformatic pipeline, we were able to detect all the mutations in the CF-positive samples. No pathogenic variants were detected in sample 1 (normal control) in both runs. Repeated samples in the inter- and intra-run analyses were found to be concordant (See technical validation section for more details).

Here, we provide the FASTQ files for each of the samples in this validation study. Tables 1 and 2 provide the coverage summary for each sample and each exon. Furthermore, in the method and technical validation section, we describe the steps and quality control (QC) performed to ensure the accuracy and precision of the assay.

To our knowledge, no previous studies have critically evaluated the sequencing performance of the Accel-Amplicon CFTR panel. As analytical performance of the methodology is vital for a clinical test, the data generated in this study can be evaluated by clinical genetic laboratories that are interested in employing the Accel-Amplicon CFTR panel to screen CF carriers. As carrier screening becomes more well-known and consumer demand increases, this method fulfils the need of an affordable and time-sensitive approach to screen *CFTR* mutations in general population carrier screening with a maximum detection rate.

## Methods

### Validation samples acquisition and DNA quantification

The following DNA samples (samples 1–3, 5–8) were obtained from the NIGMS Human Genetic Cell Repository at the Coriell Institute for Medical Research (see the corresponding Coriell naming convention in Table 3). Sample 4 was acquired from a patient; an informed consent was obtained for research using an IRB protocol (06-004886) at the Center for Applied Genomics at the Children’s Hospital of Philadelphia. The consent agreement states that genotype data may be shared with public data repositories for research purposes, and that the patient’s personal information would be kept private and unidentifiable in any publication or presentation. DNA concentration was calculated using a Qubit dsDNA HS Assay Kit (Thermo Fisher Scientific, catalogue number Q32851). Samples were diluted down to 5 ng/mL with Pre-PCR TE buffer and a final volume of 10 μL containing 20 ng input DNA was used.

**Table 3:** Sample manifest.

Sample:	Sample 1	Sample 2	Sample 3	Sample 4	Sample 5	Sample 6	Sample 7	Sample 8
*CFTR* Allele 1:	n/a	p.Arg117His	c.489 + 1G > T	p.Phe508del	p.Ile507del	c.2657 + 5G > A	p.Arg1162X	c.3528delC
*CFTR* Allele 2:	n/a	p.Phe508del	c.579 + 1G > T	n/a	n/a	c.2657 + 5G > A	n/a	p.Phe508del
Catalog Number:	NA12878	NA13591	NA11280	CF_Sample_4	NA11277	NA11859	NA12585	NA11275

### Library preparation

Library preparation was performed using the Accel-Amplicon CFTR panel (Swift Bioscience, catalogue number AL-55048) in accordance with the manufacturer’s protocol. In brief, multiplex PCR was performed on the sample DNA using the reagents provided by the Accel-Amplicon panel kit for 4 cycles of 10 sec at 98 °C, 5 min at 63 °C, 1 min at 65 °C and 22 cycles of 10 sec at 98 °C, 1 min at 64 °C. Size selection and clean-up were performed using SPRIselect beads (Beckman Coulter, catalogue number B23318) with a ratio of 1.2. Indexing sequencing adapters were then ligated to each library at 37 °C for 20 minutes. A second clean-up step was performed using SPRIselect beads at a ratio of 0.85 and rediluted with 20 mL of Post-PCR TE buffer. Quantification of adapted libraries was performed by qPCR using KAPA Library Quantification Kit (KAPA Biosystems, catalogue number 07960140001).

### Next-generation sequencing

Illumina MiSeq Nano Reagent Kit V2 was used to sequence the samples (Table 1). The final pooled concentration of 2 nM (5 μL was used) was mixed with 0.2 N NaOH (5 μL). The mixture was then mixed with 990 μL of pre-chilled HT1 to obtained a 10 pM denatured library mixed. No PhiX spike-in was used.

### Bioinformatic analysis

Sequencing data was analysed based on the bioinformatic pipeline recommended and provided by Swift Biosciences. In short, adapter-trimmed paired-end FASTQ files were generated by the Illumina MiSeq upon completion of the sequencing run (Note: adapter trimming can be done post FASTQ generation). For each sample, an alignment in Sequence Alignment Map (SAM) format was generated from the pair of FASTQ files using Burrows-Wheeler Aligner (BWA) and hg19 human genome reference. The SAM file was further modified by SAMtools to sort the file by name for Swift primerclip preparation. Due to the presence of synthetic primer sequences at the start or end of reads, the primerclip tool was used to remove these sequences before proceeding with downstream analysis. With both Picard’s AddOrReplaceReadGroups tool and SAMtools, the primer-clipped SAM file was converted to BAM format and an indexed BAM file was generated. Variant calling was performed using GATK HaplotypeCaller. To determine quality metrics at the sample and interval level, Picard’s CollectTargetPcrMetrics was used.

### Sanger sequencing

Pathogenic variants were confirmed using Sanger sequencing. PCR was performed using QIAGEN Fast Cycling PCR kit (#203743) with primers flanking the variants of interest (Tables 4 and 5). The PCR conditions were: 5 minutes at 95 °C, 35 cycles [5 seconds at 96 °C, 5 seconds at 58 °C, 40 seconds at 68 °C], 1 minute at 72 °C. PCR products were purified using Applied Biosystems ExoSAP-IT PCR Product Cleanup Reagent (#78201.1.ML). Sequencing reactions were performed using Applied Biosystems BigDye Terminator v1.1 Cycle Sequencing Kit (#4337449), and were purified using Applied Biosystem Centri-Seq. 8-Well Strips (#4367820). Sanger sequencing was performed using Applied Biosystems 3500 Genetic Analyzer (#4440462).

**Table 4:** Primer sequences for variant detection.

Primer Name	Sequences (5′ to 3′)	Variants detected
Exon 4 F	TGGCCACTATTCACTGTTTAACTT	p.Arg117His; c.489 + 1G > T
Exon 4 R	GAGGCAGTTTACAGAAGATACTCAA	
Exon 5 F	TTGAAAGAAACATTTATGAACCTGA	c.579 + 1G > T
Exon 5 R	CTATTATCTGACCCAGGAAAACTC	
Exon 10 F	CACTTCTGCTTAGGATGATAATTGG	p.Ile507del; p.Phe508del
Exon 10 R	CAGTAGCTTACCCATAGAGGAAACA	
Exon 14b F	CAGGAACACAAAGCAAAGGAA	c.2657 + 5G > A
Exon 14b R	CAGGAATGTGTCACCTCACC	
Exon 19 F	TGAAAAGCCCGACAAATAACC	p.Arg1162X; c.3528delC
Exon 19 R	ACTTGTTTGGCAGAATGGAAC

**Table 5:** Sample file names as listed in SRA.

Sample	Run1	Run2
1	SRR8945290_1_1.fastq	SRR10164005_1_1.fastq
	SRR8945290_1_2.fastq	SRR10164005_1_2.fastq
2	SRR8945291_3_1.fastq	SRR8945291_2_1.fastq
	SRR8945291_3_2.fastq	SRR8945291_2_2.fastq
3	SRR8945292_4_1.fastq	SRR8945292_6_1.fastq
	SRR8945292_4_2.fastq	SRR8945292_6_2.fastq
4	SRR8945293_2_1.fastq	SRR8945293_3_1.fastq
	SRR8945293_2_2.fastq	SRR8945293_3_2.fastq
		SRR8945293_4_1.fastq
		SRR8945293_4_2.fastq
		SRR8945293_5_1.fastq
		SRR8945293_5_2.fastq
5		SRR8945286_7_1.fastq
		SRR8945286_7_2.fastq
6		SRR8945287_8_1.fastq
		SRR8945287_8_2.fastq
7		SRR8945288_9_1.fastq
		SRR8945288_9_2.fastq
8		SRR8945289_10_1.fastq
		SRR8945289_10_2.fastq

#### BWA-MEM alignment

bwa mem ${FASTA} ${Sample_ID}_R1.fastq.gz ${Sample_ID}_R2.fastq.gz -U 17 -M -t 32 > ${Sample_ID}_bwa.sam.

#### SAMtools sort SAM

samtools sort -n ${Sample_ID}_bwa.sam -o ${Sample_ID}_bwa_nsorted.sam.

#### Primerclip

primerclip Accel-Amplicon_CFTR_masterfile.txt ${Sample_ID}_bwa_nsorted.sam ${Sample_ID}_bwa_primertrimmed.sam.

#### SAMtools convert SAM to BAM

java -jar picard.jar AddOrReplaceReadGroups I=${Sample_ID}_bwa_primertrimmed.sam O=${Sample_ID}_bwa_primertrimmed.bam SO=coordinate RGID=snpID LB=swift SM=${Sample_ID} PL=illumina PU=miseq VALIDATION_STRINGENCY=STRICT.

samtools index ${Sample_ID}_bwa_primertrimmed.bam ${Sample_ID}_bwa_primertrimmed.bam.bai.

#### Picard CollectPcrMetrics tool

samtools view -H ${Sample_ID}_bwa_primertrimmed.bam > ${Sample_ID}_bwa_header.txt.

cat ${Sample_ID}_bwa_header.txt cftr_180313_nonmerged_targets_5col.bed > ${Sample_ID}_bwa_fullintervals.

cat ${Sample_ID}_bwa_header.txt cftr_180313_nonmerged_targets_5col.bed > ${Sample_ID}_bwa_noprimerintervals.

java -jar picard.jar CollectTargetedPcrMetrics I=${Sample_ID}_bwa_primertrimmed.bam O=${Sample_ID}_bwa_targetPCRmetrics.txt AI=${Sample_ID}_bwa_fullintervals TI=${Sample_ID}_bwa_noprimerintervals R=${FASTA} PER_TARGET_COVERAGE=${Sample_ID}_bwa_perTargetCov.txt VALIDATION_STRINGENCY=STRICT.

#### GATK variant calling

java -jar GenomeAnalysisTK.jar -T HaplotypeCaller -R ${FASTA} -I ${Sample_ID}_bwa_primertrimmed.bam -stand_call_conf 20 -stand_emit_conf 20 -mbq 20 -L CFTR_merged_5col.bed -o ${Sample_ID}_bwa_gatkHC.vcf.

## Data Records

There are eight unique samples in our cohort. Samples 1–4 were analysed in both runs. Samples 5–8 were analysed in run 2. Sample 4 was run in triplicate in the second run. fastq can be accessed from the Sequence Read Archive (SRA) repository under SRA: SRP193469^13^. Direct FASTQ files can be downloaded via SRA Toolkit using command line “fastq-dump–split-3 -G SRR#” (Table 5). BAM files can be downloaded at (10.6084/m9.figshare.11341958.v1), and VCF files can be downloaded at (10.6084/m9.figshare.10565513.v1)^14,15^.

## Technical Validation

### Library quantitation

To evaluate whether the DNA samples were successfully processed using this Swift Accel Amplicon protocol, we used the KAPA Library Quantification Kit to measure the library concentration. During qPCR, primers bound to the Illumina P5 and P7 flow cell oligo sequences and the concentrations of the samples were assessed by measuring the SYBR green fluorescence intensity; this method specifically measures the adapted DNA, excluding any unadapted DNA fragments generated during the PCR step. The concentration of each sample in both runs are listed in Table 6.

**Table 6:** Sequencing quality assessment.

Run	Sample	Concentration (nM)	Cluster Density (k/mm^2^)	% Q30
1	Sample 1	5.6	807 ± 1	98.08
1	Sample 2	4.5		
1	Sample 3	6.7		
1	Sample 4	2.6		
2	Sample 1	16.5	534 ± 8	98.05
2	Sample 2	14.6		
2	Sample 3	14.2		
2	Sample 4-1	14.5		
2	Sample 4-2	16.9		
2	Sample 4-3	16.0		
2	Sample 5	15.0		
2	Sample 6	19.1		
2	Sample 7	10.7		
2	Sample 8	12.5		

### Sequencing data assessment

**Fig. 2:**
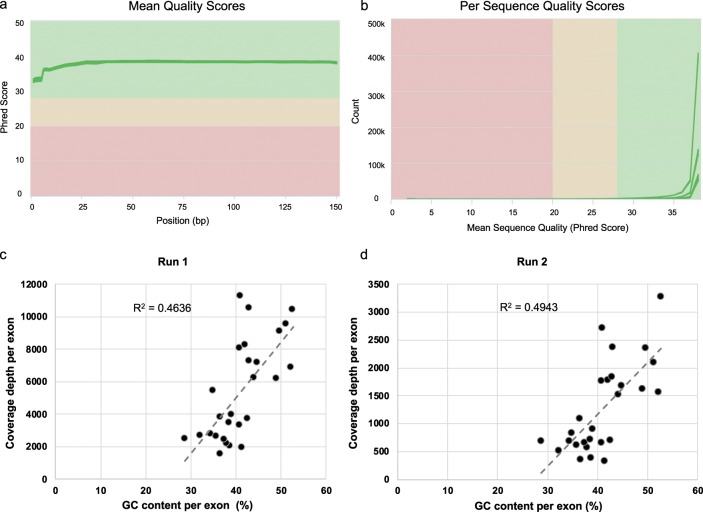
Sequence quality and coverage depth per exon. Sequence quality was assessed using MultiQC. Each green line represents one FASTQ file. (**a**) Mean quality value across each base position in the read. (**b**) Number of reads with average quality score. (**c**,**d**) For both runs, the coverage depth of exons increases as the GC content approaches 50%.

Pooled libraries were sequenced using Illumina MiSeq Nano Reagent Kit V2 kit (300 cycles). The cluster densities for run 1 and 2 were 807 ± 1 k/mm^2^ and 534 ± 8 k/mm^2^, with 98.08% and 98.05% of reads of Q30 score or more, respectively (Table 6). Further analyses of the FASTQ files using MultiQC showed that the majority of the base positions had mean quality value of Q38, while the first five bases of reads have lower quality scores (at around Q33) (Fig. 2a). For all FASTQ files, the majority of the reads had quality value of Q38 (Fig. 2b)^16^. Overall coverage depth of all processed samples is demonstrated in Table 1. As expected, the mean coverage depth in run 1 (5753x) is higher than those of run 2 (1344x), as there are fewer samples pooled into one flow cell in run 1 (Table 1). Moreover, all samples from run 1 have 100% of regions with more than 20x coverage depth (Table 1). For run 2, all samples have less than 20x coverage at the 3′UTR region (chr7:117308320–117308346; CFTR:c.*1158_*1184). This region has no known pathogenic variants described in HGMD or in ClinVar. In addition, samples 5, 6, and 7 have no coverage for two bases in intron 8 (chr7:117188661–117188662; CFTR:c.1210-13_1210-12). This is a common TG repeat deletion that is present in 22.92% of general population according to gnomAD. Next, we assessed the coverage depth per exon, and investigated the inter-exonic depth variability (Tables 2 and 7). We found that the coverage depth was higher as the GC content of the exon was closer to 50% for both runs (Fig. 2c,d). As expected for amplicon sequencing, the majority of sequencing reads (98–99%) were aligned to the targeted regions (See Supplementary File 1 for BED file).

**Table 7:** Sequencing coverage depth per exon for each sample.

Run:	1	1	1	1	2	2	2	2	2	2	2	2	2	2
Swift exon annotation	Sample 1	Sample 2	Sample 3	Sample 4	Sample 1	Sample 2	Sample 3	Sample 4	Sample 4	Sample 4	Sample 5	Sample 6	Sample 7	Sample 8
5′UTR/exon 1	3728	4312	4004	12623	1193	898	1953	1729	1520	1868	1886	1662	1902	1525
intron 1	1424	1235	1912	4771	1011	734	1584	1228	1261	1426	1415	1235	1368	1146
exon 2	1367	1359	958	4068	208	181	395	352	331	463	289	311	287	363
exon 3	1737	2150	1536	5175	476	382	705	636	628	761	637	653	616	597
exon 4	6836	6943	6411	21902	1756	1295	2865	2555	2365	2920	2630	2403	2596	2312
exon 5	1857	1912	1523	5914	508	398	756	766	739	944	627	636	685	713
exon 6	6477	6372	5699	19662	1524	1136	2502	2250	2146	2730	2139	2063	2221	2242
exon 7	2590	2258	2304	8208	758	616	1338	1149	1215	1435	1074	1106	1044	1136
exon 8	4109	4346	3786	12778	1158	824	1827	1703	1540	1848	1622	1603	1657	1393
exon 9	1061	1038	959	3174	264	204	477	441	351	479	334	347	316	304
exon 10	1384	1664	1217	3817	283	192	451	442	423	469	374	412	416	307
exon 11	2434	2512	1864	7022	547	394	828	840	807	907	712	634	741	664
exon 12	5806	5738	4783	16703	1232	1026	2202	1874	1788	2446	1716	1773	1776	1974
intron 12	1404	1434	1496	3916	573	415	884	797	708	901	750	775	768	726
exon 13	1703	1747	1582	4855	511	346	872	786	695	857	674	663	703	746
exon 14	5507	5997	5398	15272	1288	1010	2256	1799	1660	1975	1987	1851	1998	1880
exon 15	1551	1670	1370	4210	417	324	747	625	609	699	570	587	604	536
exon 16	7075	6191	7432	21016	2308	2028	4277	3478	3283	3984	3353	2909	3462	3600
exon 17	7778	8137	7004	22096	2029	1542	3488	2881	2551	3103	2926	2777	3021	2763
exon 18	1652	1681	1485	4868	488	371	757	735	691	824	667	676	704	621
exon 19	2659	2826	2117	8174	646	520	1082	1031	896	1029	971	885	1021	930
exon 20	2298	2385	1994	6595	461	382	812	748	686	825	635	644	685	725
exon 21	2556	2750	1969	7690	490	367	798	788	731	888	632	697	651	871
exon 22	4749	5051	4783	14477	1400	1088	2248	2067	1989	2156	1841	1878	1949	1689
intron 22	2844	2607	3360	9895	1500	1117	2281	2157	1908	2354	1980	1826	1954	1641
exon 23	4620	4976	4325	14794	1304	972	2103	1932	1766	2061	1702	1607	1691	1575
exon 24	1739	2126	1533	5327	385	302	615	568	521	659	515	485	543	511
exon 25/intron 25	5922	6158	5744	18482	1761	1318	2992	2695	2338	2906	2521	2223	2468	2266
exon 26	3775	4040	3041	10884	626	458	988	1038	969	1152	730	799	780	774
exon 27/3′UTR	4555	4687	4425	13837	1126	896	2009	1702	1560	1925	1602	1527	1662	1539

### Assay validation of CF-positive samples

**Fig. 3:**
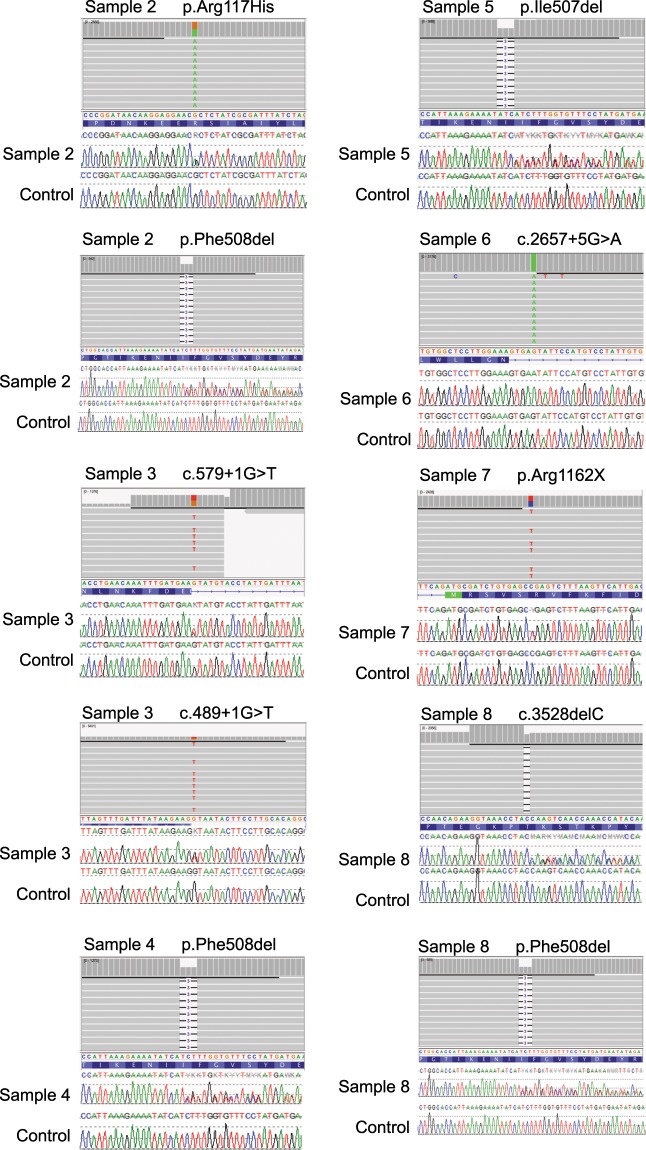
Variant visualization using IGV and Mutation Surveyor. The variants for each corresponding sample are confirmed by visualizing the BAM files in Integrative Genomic Viewer (IGV). The Sanger sequence traces visualized using MutationSurveyor are also shown for each variant of each sample.

Samples used in this validation study have known pathogenic *CFTR* mutations (Table 3), and they were used to validate this Swift Accel-Amplicon CFTR Panel for usage in a clinical laboratory setting. Analytical validation is a vital component in the process of launching a clinical genetic test, as it demonstrates the quality and performance of the testing method and the accuracy of the assay result. Here, we evaluate the capability of this assay by assessing the variants that were detected in each sample. As expected, there were no pathogenic variants detected in the control sample (sample 1) for both runs. The pathogenic variants of samples 2–8 were confirmed by the manufacturer-recommended bioinformatic pipeline. These genotypes can be visualized using Integrative Genome Viewer (IGV), and they have also been confirmed using Sanger sequencing (Fig. 3); this yields a 100% sensitivity. Furthermore, samples 1–4 were sequenced in both runs, and sample 4 was sequenced three times in run 2. All results were concordant and matched to the referenced genotypes, hence the repeatability and reproducibility is 100%. Additionally, since there can be non-pathogenic variants in *CFTR*, we provide a table of all the variants detected in each VCF file for each sample in both run (Online-only Table 1). HGVS nomenclature and GnomAD frequencies for each variant are also listed. Of note, the VCF for sample 1 in run 2 contains a variant that is not present in run 1. This variant is a common two-nucleotide deletion of a TG-repeat stretch in intron 8. This dinucleotide repeat is adjacent to a poly-T stretch that also has common deletions and duplications. This discrepancy may be due to the fact that NGS alignment and annotation tools cannot reliably detect small insertions/deletions at repetitive regions. Sanger sequencing is still the preferred method to reliably detect variants at this repeat.

### Supplementary information

Supplementary File 1

## Data Availability

Swift Primerclip installation instructions, scripts, and examples can be found at https://github.com/swiftbiosciences/primerclip. Current available methods for downloading the Swift Primerclip tool are a pre-compiled binary for linux on x86_64 and building from source using Haskell-stack build tool. Additional requirements include SAMTools (1.6-2-gf068ac2), Picard Tools (2.1.0), BWA (0.7.17-r1188), GATK (3.5-0-g36282e4), and Java (1.8). Codes and parameters are described as below.

## Online-only Table

**Online-only Table 1:** Variants listed in each VCF file for each sample.

Run	Sample	Chrom	POS	ID	Ref	Alt	Nomenclature	Gnomad Freq
1	2	7	117120145	.	G	C	c.-4G > C	0.00007433
1	2	7	117171029	.	G	A	c.350G > A	0.001438
1	2	7	117176568	.	AGATT	A	c.744-9_744-6del	0.2648
1	2	7	117176738	.	C	T	c.869 + 11C > T	0.06933
1	2	7	117188682	.	G	T	c.1210-13G > T	0.0752
1	2	7	117188684	.	T	G	c.1210-11T > G	0.008495
1	2	7	117199457	.	A	G	c.1393-61A > G	0.267
1	2	7	117199533	.	G	A	c.1408G > A	0.4865
1	2	7	117199644	.	ATCT	A	c.1521_1523del	0.007172
1	2	7	117229537	.	T	A	c.1680-870T > A	0.5654
1	3	7	117171169	.	G	T	c.489 + 1G > T	0.00006857
1	3	7	117174420	.	G	T	c.579 + 1G > T	0.0000462
1	3	7	117176568	.	AGATT	A	c.744-9_744-6del	0.2648
1	3	7	117176738	.	C	T	c.869 + 11C > T	0.06933
1	3	7	117188682	.	G	T	c.1210-13G > T	0.0752
1	3	7	117199457	.	A	G	c.1393-61A > G	0.267
1	3	7	117199533	.	G	A	c.1408G > A	0.4865
1	3	7	117229537	.	T	A	c.1680-870T > A	0.5654
1	3	7	117307286	.	GT	G	c.*133del	0.4942
1	4	7	117176568	.	AGATT	A	c.744-9_744-6del	0.2648
1	4	7	117176738	.	C	T	c.869 + 11C > T	0.06933
1	4	7	117188682	.	G	T	c.1210-13G > T	0.0752
1	4	7	117199457	.	A	G	c.1393-61A > G	0.267
1	4	7	117199533	.	G	A	c.1408G > A	0.4865
1	4	7	117199644	.	ATCT	A	c.1521_1523del	0.007172
1	4	7	117229537	.	T	A	c.1680-870T > A	0.5654
1	4	7	117246636	.	G	A	c.2909-92G > A	0.1755
1	4	7	117307108	.	G	A	c.4389G > A	0.2206
1	4	7	117307286	.	GT	G	c.*133del	0.4942
1	4	7	117308413	.	C	T	c.*1251C > T	0.2566
2	1	7	117188660	.	ATG	A	c.1210-13_1210-12del	0.2292
2	2	7	117120145	.	G	C	c.-4G > C	0.00007433
2	2	7	117171029	.	G	A	c.350G > A	0.001438
2	2	7	117176568	.	AGATT	A	c.744-9_744-6del	0.2648
2	2	7	117176738	.	C	T	c.869 + 11C > T	0.06933
2	2	7	117188682	.	G	T	c.1210-13G > T	0.0752
2	2	7	117188684	.	T	G	c.1210-11T > G	0.008495
2	2	7	117199457	.	A	G	c.1393-61A > G	0.267
2	2	7	117199533	.	G	A	c.1408G > A	0.4865
2	2	7	117199644	.	ATCT	A	c.1521_1523del	0.007172
2	2	7	117229537	.	T	A	c.1680-870T > A	0.5654
2	3	7	117171169	.	G	T	c.489 + 1G > T	0.00006857
2	3	7	117174420	.	G	T	c.579 + 1G > T	0.0000462
2	3	7	117176568	.	AGATT	A	c.744-9_744-6del	0.2648
2	3	7	117176738	.	C	T	c.869 + 11C > T	0.06933
2	3	7	117188682	.	G	T	c.1210-13G > T	0.0752
2	3	7	117199457	.	A	G	c.1393-61A > G	0.267
2	3	7	117199533	.	G	A	c.1408G > A	0.4865
2	3	7	117229537	.	T	A	c.1680-870T > A	0.5654
2	3	7	117307286	.	GT	G	c.*133del	0.4942
2	4-1	7	117176568	.	AGATT	A	c.744-9_744-6del	0.2648
2	4-1	7	117176738	.	C	T	c.869 + 11C > T	0.06933
2	4-1	7	117188682	.	G	T	c.1210-13G > T	0.0752
2	4-1	7	117199457	.	A	G	c.1393-61A > G	0.267
2	4-1	7	117199533	.	G	A	c.1408G > A	0.4865
2	4-1	7	117199644	.	ATCT	A	c.1521_1523del	0.007172
2	4-1	7	117229537	.	T	A	c.1680-870T > A	0.5654
2	4-1	7	117246636	.	G	A	c.2909-92G > A	0.1755
2	4-1	7	117307108	.	G	A	c.4389G > A	0.2206
2	4-1	7	117307286	.	GT	G	c.*133del	0.4942
2	4-1	7	117308413	.	C	T	c.*1251C > T	0.2566
2	4-2	7	117176568	.	AGATT	A	c.744-9_744-6del	0.2648
2	4-2	7	117176738	.	C	T	c.869 + 11C > T	0.06933
2	4-2	7	117188682	.	G	T	c.1210-13G > T	0.0752
2	4-2	7	117199457	.	A	G	c.1393-61A > G	0.267
2	4-2	7	117199533	.	G	A	c.1408G > A	0.4865
2	4-2	7	117199644	.	ATCT	A	c.1521_1523del	0.007172
2	4-2	7	117229537	.	T	A	c.1680-870T > A	0.5654
2	4-2	7	117246636	.	G	A	c.2909-92G > A	0.1755
2	4-2	7	117307108	.	G	A	c.4389G > A	0.2206
2	4-2	7	117307286	.	GT	G	c.*133del	0.4942
2	4-2	7	117308413	.	C	T	c.*1251C > T	0.2566
2	4-3	7	117176568	.	AGATT	A	c.744-9_744-6del	0.2648
2	4-3	7	117176738	.	C	T	c.869 + 11C > T	0.06933
2	4-3	7	117188682	.	G	T	c.1210-13G > T	0.0752
2	4-3	7	117199457	.	A	G	c.1393-61A > G	0.267
2	4-3	7	117199533	.	G	A	c.1408G > A	0.4865
2	4-3	7	117199644	.	ATCT	A	c.1521_1523del	0.007172
2	4-3	7	117229537	.	T	A	c.1680-870T > A	0.5654
2	4-3	7	117246636	.	G	A	c.2909-92G > A	0.1755
2	4-3	7	117307108	.	G	A	c.4389G > A	0.2206
2	4-3	7	117307286	.	GT	G	c.*133del	0.4942
2	4-3	7	117308413	.	C	T	c.*1251C > T	0.2566
2	5	7	117188660	.	ATGTG	A,ATG	c.1210-13_1210-12del	0.2292
2	5	7	117199533	.	G	A	c.1408G > A	0.4865
2	5	7	117199640	.	TATC	T	c.1519_1521del	0.00004246
2	5	7	117229536	.	A	G	c.1680-871A > G	0.01043
2	5	7	117229537	.	T	A	c.1680-870T > A	0.5654
2	5	7	117235055	.	T	G	c.2562T > G	0.3901
2	5	7	117246636	.	G	A	c.2909-92G > A	0.1755
2	5	7	117307108	.	G	A	c.4389G > A	0.2206
2	5	7	117307286	.	GT	G	c.*133delT	0.4942
2	5	7	117308413	.	C	T	c.*1251C > T	0.2566
2	6	7	117188660	.	ATGTG	A,ATG	c.1210-13_1210-12del	0.2292
2	6	7	117199533	.	G	A	c.1408G > A	0.4865
2	6	7	117229537	.	T	A	c.1680-870T > A	0.5654
2	6	7	117235055	.	T	G	c.2562T > G	0.3901
2	6	7	117242922	.	G	A	c.2657 + 5 G > A	0.0000707
2	6	7	117246636	.	G	A	c.2909-92 G > A	0.1755
2	6	7	117307108	.	G	A	c.4389G > A	0.2206
2	6	7	117307286	.	GT	G	c.*133delT	0.4942
2	6	7	117308413	.	C	T	c.*1251C > T	0.2566
2	7	7	117188660	.	ATG	A	c.1210-13_1210-12del	0.2292
2	7	7	117199533	.	G	A	c.1408G > A	0.4865
2	7	7	117229537	.	T	A	c.1680-870T > A	0.5654
2	7	7	117235055	.	T	G	c.2562T > G	0.3901
2	7	7	117246636	.	G	A	c.2909-92G > A	0.1755
2	7	7	117267591	.	C	T	c.3484C > T	0.00005674
2	7	7	117307108	.	G	A	c.4389G > A	0.2206
2	7	7	117307286	.	GT	G	c.*133del	0.4942
2	7	7	117308413	.	C	T	c.*1251C > T	0.2566
2	8	7	117176568	.	AGATT	A	c.744-9_744-6del	0.2648
2	8	7	117176738	.	C	T	c.869 + 11C > T	0.06933
2	8	7	117188682	.	G	T	c.869 + 11C > T	0.06933
2	8	7	117199457	.	A	G	c.1393-61A > G	0.267
2	8	7	117199533	.	G	A	c.1408G > A	0.4865
2	8	7	117199644	.	ATCT	A	c.1521_1523del	0.007172
2	8	7	117229537	.	T	A	c.1680-87 T > A	0.5654
2	8	7	117267633	.	AC	A	c.3528del	0.0001559
